# Automated triage of cancer-suspicious skin lesions with 3D total-body photography

**DOI:** 10.1038/s41746-025-02070-7

**Published:** 2025-11-21

**Authors:** Nicholas R. Kurtansky, Maura C. Gillis, Noel C. F. Codella, Brian M. D’Alessandro, Zongyuan Ge, Pascale Guitera, Allan C. Halpern, Harald Kittler, Josep Malvehy, Konstantinos Liopyris, Victoria J. Mar, Linda K. Martin, Lara Valeska Maul, Alexander Navarini, Tarlia Rajeswaran, Vin Rajeswaran, Nadia Reichman, H. Peter Soyer, Jochen Weber, Siyuan Yan, Veronica Rotemberg, Kivanc Kose

**Affiliations:** 1 Dermatology Service, Department of Medicine, Memorial Sloan Kettering Cancer Center, New York, NY USA; 2 Microsoft, Redmond, WA USA; 3 Canfield Scientific, Inc., Parsippany, NJ USA; 4 Department of Data Science and AI, Monash University, Melbourne, VIC Australia; 5 AIM for Health Lab, Faculty of IT, Monash University, Melbourne, VIC Australia; 6 Melanoma Institute Australia, Sydney, NSW Australia; 7 Faculty of Medicine and Health, The University of Sydney, Sydney, NSW Australia; 8 Sydney Melanoma Diagnostic Centre, Royal Prince Alfred Hospital, Camperdown, NSW Australia; 9 ViDIR Group, Department of Dermatology, Medical University of Vienna, Vienna, Austria; 10 Dermatology Service, Melanoma Unit, Hospital Clínic de Barcelona, IDIBAPS, Universitat de Barcelona, ITOBOS, Barcelona, Spain; 11 Centro de Investigación Biomédica en Red de Enfermedades Raras (CIBER ER), Instituto de Salud Carlos III, Barcelona, Spain; 12 University of Athens Medical School, Athens, Greece; 13 Victorian Melanoma Service, Alfred Health, Melbourne, VIC Australia; 14 School of Public Health and Preventive Medicine, Monash University, Melbourne, VIC Australia; 15 School of Clinical Medicine, Faculty of Medicine & Health, University of New South Wales, Sydney, NSW Australia; 16 Department of Dermatology, University Hospital of Zurich, Zurich, Switzerland; 17 Faculty of Medicine, University of Zurich, Zurich, Switzerland; 18 Department of Dermatology, University Hospital of Basel, Basel, Switzerland; 19 FNQH Cairns Integrated Melanoma Centre, Cairns, QLD Australia; 20 Frazer Institute, The University of Queensland, Dermatology Research Centre, Brisbane, QLD Australia; 21 Faculty of Engineering, Monash University, Melbourne, VIC Australia

**Keywords:** Cancer, Computational biology and bioinformatics, Diseases, Health care, Medical research, Oncology

## Abstract

Careful selection of skin lesions that require expert evaluation is important for early skin cancer detection. Yet challenges include lack of cost-effective asymptomatic screening, geographical inequality in access to specialty dermatology, and long wait times due to exam inefficiencies and staff shortages. Machine learning models trained on high-quality dermoscopy photos have been shown to aid clinicians in diagnosing individual, hand-selected skin lesions. In contrast, models designed for triage have been less explored due to limited datasets that represent a broader net of skin lesions. 3D total body photography is an emerging technology used in dermatology to document all apparent skin lesions on a patient for skin cancer monitoring. A multi-institutional and global project collected over 900,000 lesion crops off 3D total body photos for an online grand challenge in machine learning. Here we summarize the results of the competition, ‘ISIC 2024 – Skin Cancer Detection with 3D-TBP’, demonstrate superiority of a model that utilized intra-patient context against a prior published approach, and explore clinical plausibility of automated atypical skin lesion triage through an ablation study.

## Introduction

Applications of machine learning (ML) and artificial intelligence in dermatologic imaging have shown substantial promise in skin cancer classification. Existing lesion-based approaches appear to improve clinical decision-making^1^ in certain usages and outperform dermatologists in theoretical settings^2–7^. Thus far, diagnostic accuracies of ML-models have only been evaluated over pre-selected lesions, which typically require a human, often an expert clinician or physician assistant (e.g., melanographer), to identify lesions of interest for dermoscopic evaluation and ancillary testing^8,9^. This presents logistical challenges that may lead to overlooked malignancies, and resulting datasets could be hampered by selection bias, failing to represent more commonly occurring lesions for broader applicability.

In contrast to lesion-based approaches, applications of ML-assisted triage have been less explored. High-risk patients often present with hundreds of lesions that undergo clinical examination by a dermatologist^10–12^. Machine-guided identification of atypical lesions requiring expert evaluation may improve the efficiency and sensitivity of full-body skin examination workflows and lead to a more effective health system approach to skin surveillance. If accessible, triage applications may enhance the selection of at-risk lesions and corresponding individuals for specialist referral, benefit underserved and understaffed populations, and improve early detection^13–16^, an important factor in long-term patient outcomes^17,18^.

ML applications for dermatologic triage ideally should consider all lesions from an individual. Notable datasets used to develop high-performing dermoscopy-based ML-models are typically constructed of dermoscopy images from clinical practice, which skew towards lesions of heightened clinical concern. While resulting models can constructively provide diagnostic aid, such as advising clinicians during biopsy decisions, they may generalize poorly to triage tasks like atypical lesion identification prior to clinical and dermoscopic evaluation.

Innovations in total-body photography (TBP) imaging offer a possible approach to ML-assisted triage by minimizing lesion selection bias and improving the efficiency of comprehensive skin image analysis^19^. The Vectra WB360 is a 3-dimensional (3D) TBP imaging system (Canfield Scientific, Inc.) that uses an array of stereoscopically positioned camera pairs in a fixed apparatus to capture a patient’s complete visible cutaneous surface area in one macro-quality-resolution 3D avatar. The images are standardized with respect to lighting and camera position, and their capture is virtually instantaneous. Dermatologists primarily use TBP images to monitor patients with complex phenotypes and identify new or changing pigmented skin lesions through visual comparison between a prior image and the patient in the clinic^20^. The Vectra WB360 system is equipped with a proprietary ML-model that can identify unobstructed lesions^21,22^ on the patient and characterize each according to various properties related to color, shape, and size (hereafter referred to as *WB360-measurements*). Each lesion identified by the software can be exported as an individual image (hereafter referred to as a *tile*)^23^.

A dataset composed of the lesion tiles from a patient sample, with minimal pre-selection, has several advantages. First of all, the standard narrow field-of-view of the tiles (15 × 15 mm) provides a solution for developing open-source 3D TBP applications while maintaining patient privacy and confidentiality^24–26^. Additionally, 3D TBP offers an effective way to account for all lesions in a patient, except those located on soles of feet or obstructed by hair or clothing. Therefore, it facilitates quick data gathering while mitigating potential lesion selection bias. Lastly, the pixel resolution of tiles simulates clinical overview photography and smartphone imaging, which are widely used in non-specialist clinical practice (e.g., primary care), and which may be helpful in training more comprehensive ML-models.

A critical question is whether tiles contain enough information for clinical diagnostic decisions despite being lower quality than lesion-based imaging, such as dermoscopy. A recent pilot study by *Marchetti* et al. reported that regression models could use WB360-measurments extracted from lesion tiles, like color variation, size, and border irregularity, to reduce the number of lesions requiring close inspection by 75% while maintaining 95% sensitivity for melanoma^27^. To date, it is the only prior study published in the area of automated 3D TBP-based skin cancer detection. Though promising, the model has not been validated in further studies or in data from other centers, and it is plausible that more complex ML-based approaches could perform better. In this regard, the practical impact of automated 3D TBP-based triage remains to be tested. Additionally, the source of predictive power from 3D TBP images is not well understood because tile images are lower in resolution and contain less morphological information than dermoscopic images.

The International Skin Imaging Collaboration (ISIC) has been influential in the field of dermatologic image analysis since 2016. ISIC has published extensive datasets and has organized competitions to engage computer vision experts in developing ML-models that perform tasks relevant to skin cancer detection and classification^28–30^. The ISIC 2024 competition (hereafter referred to as *ISIC’24*) focused on TBP imaging, reflecting real-world scenarios of identifying malignant lesions among all lesions from a diverse set of patients^23,31^.

This study analyzes the results of ISIC’24 against the previously reported model (hereafter referred to as *Marchetti* et al.)^27^ and conducts an ablation study using the competition winner to understand how various information types (i.e., tiles and metadata describing demographics, appearance, and patient-context) affect model performance. The four input information classes are defined in Table 1. The study had three primary objectives regarding the application of ML to 3D TBP: (1) To assess if automated lesion selection could accurately support total-body skin examinations. (2) To identify characteristics that explain risk perception among ML-based 3D TBP algorithms. (3) Finally, to assess the relative importance of different input features on diagnostic performance.

**Table 1:** Description of input feature classes

Four information classes			
Tiles	Basic “demographics” metadata	WB360 “appearance” metadata	Patient contextual information
Image files. Lesions are cropped from Vectra WB360 photos and saved in jpeg format.	Image metadata that are known with basic knowledge of the patient/lesion and do not require measurements by the Vectra WB360 system. Namely, ● Patient age ● Patient sex ● Anatomic location ● Hospital/institution These are common in dermatology datasets of other imaging modalities, such as dermoscopy.	Image metadata that is derived through Vectra WB360 tooling. For example, ● Lesion area ● Color contrast ● Border irregularity ● Lighting modality For full list of terms, see full data descriptor published by Kurtansky et al.^23^.	Defined broadly to include any engineered data element that describes a lesion in the context of others on the patient. For example, ● Total lesion count ● Lesion area normalized to the mean of a given patient Each of these calculations involve knowledge of other lesions on the patient using the ‘patient_id‘ attribute.

## Results

### Overview of ISIC’24 dataset and participation

ISIC’24 utilized distinct datasets for training^23^, real-time validation, and official scoring. The Methods section further details the training set, public “validation” leaderboard set, and private “evaluation” leaderboard set, which shared similar class distributions as shown in Table 2. Unless otherwise stated, all analyses are based on the private leaderboard set, containing 370,704 lesions from 935 patients; 243 was the median number of lesions per patient (*Q*_1_ = 109.5, *Q*_3_ = 491.5); the diseased class contained 342 lesions, including 99 melanomas (55 in-situ), 190 basal cell carcinomas (BCCs), and 53 squamous cell carcinomas (SCCs).

**Table 2:** Training and test set distributional comparisons

	Training		Public LB subset		Private LB subset	
**Total patients**	**1042**	**100%**	**342**	**100%**	**935**	**100%**
Tile-images per patient						
<100	221	21.2%	89	26.0%	213	22.8%
100–199	225	21.6%	58	17.0%	177	18.9%
200–299	157	15.1%	44	12.9%	143	15.3%
300–399	112	10.7%	35	10.2%	102	10.9%
400+	327	31.4%	116	33.9%	300	32.1%
Malignant labels per patient						
None	783	75.1%	245	71.6%	698	74.7%
Exactly one	193	18.5%	74	21.6%	183	19.6%
More than one	66	6.3%	23	6.7%	54	5.8%
Patients by source						
*Australia*						
ACEMID MIA	44	4.2%	13	3.8%	34	3.6%
FNQH Cairns	0	0.0%	74	21.6%	177	18.9%
Monash University and Alfred Health	0	0.0%	11	3.2%	30	3.2%
University of Queensland	176	16.9%	50	14.6%	134	14.3%
*Europe*						
Hospital Clinic de Barcelona	163	15.6%	47	13.7%	135	14.4%
Medical University of Vienna	15	1.4%	4	1.2%	12	1.3%
University Hospital of Basel	230	22.1%	51	14.9%	203	21.7%
University of Athens	16	1.5%	3	0.9%	11	1.2%
*Americas*						
Memorial Sloan Kettering Cancer Center	398	38.2%	89	26.0%	199	21.3%
**Total lesion-images**	**401,059**	**100%**	**140,770**	**100%**	**370,704**	**100%**
Label class						
Malignant	393	0.1%	138	0.1%	342	0.1%
Benign/indeterminate	400,666	99.9%	140,632	99.9%	370,362	99.9%
Label subclass						
*Malignant*						
Melanoma	157	0.0%	46	0.0%	99	0.0%
Basal cell carcinoma	163	0.0%	190	0.1%	190	0.1%
Squamous cell carcinoma	73	0.0%	53	0.0%	53	0.0%
*Benign/indeterminate, biopsied*						
Nevus	443	0.1%	116	0.1%	357	0.1%
Actinic keratosis	39	0.0%	15	0.0%	35	0.0%
Indeterminate melanocytic	75	0.0%	15	0.0%	57	0.0%
Other	118	0.0%	53	0.0%	125	0.0%
*Benign/indeterminate, not biopsied*						
NOS	399,991	99.7%	140,433	99.8%	369,788	99.8%

**Fig. 1:**
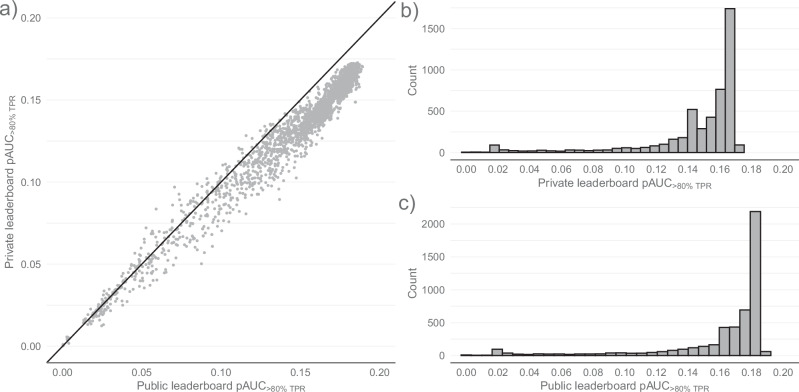
Public and private leaderboard frequency and bivariate distributions. **a** Shows a scatterplot of submissions according to their scores on the public (*x*-axis) and private (*y*-axis) leaderboards. While participant scores on the public and private leaderboard were highly correlated (*Pearson*′*s correlation coefficient* = 0.988), the deviation from the line of perfect concordance suggests some overfitting occurred. **b** Presents a histogram of scores on the private leaderboard. **c** Presents a histogram of scores on the public leaderboard.

A total of 2739 participating teams entered 4998 submissions for official scoring. Focusing on thresholds with low false negative rates required in the clinical setting, the primary metric for leaderboard scoring was partial area under the receiver operating characteristic (ROC) curve above 80% sensitivity (*pAUC*_>80% *TPR*_ ∈ [0,0.2]). Teams in the top five were awarded prizes. Winners were based in Georgia, Japan, Türkiye, Vietnam, and the United States, with scores separated by less than 0.001. ISIC’24 was highly competitive, indicated by a left-skew in both public and private leaderboards (Fig. 1) with interquartile ranges of 0.005 (*Q*_1_ = 0.1841, *Q*_3_ = 0.1893) and 0.006 (*Q*_1_ = 0.1669, *Q*_3_ = 0.1726), respectively. 46% of official submissions scored above 0.16 on the private leaderboard, with the top 1% exceeding 0.17. The five leaderboard prize winners jumped 30, 23, 764, 1137, and 1100 places from their public leaderboard standing. Successful teams tended to submit dozens to several hundreds of entries for real-time “validation” scoring. This led to a degree of overfitting, as 95.5% of official submissions scored higher on the public leaderboard than on the private leaderboard.

### Feasibility of skin cancer detection with 3D TBP-based ML-models

Measures of diagnostic classification accuracy are reported in Table 3. The winning model scored *pAUC*_>80% *TPR*_ = 0.1726 on the private leaderboard with an area under the full ROC curve (*AUC*) = 0.9668 for differentiating skin cancer. Precision is reported as the average number of lesions needing to be treated to detect a true positive (*NNT*_*x*% *SE*_). The winning model realized *NNT*_80% *SE*_ = 51.57 and *NNT*_90% *SE*_ = 98.20, meaning one true positive for every 52 and 98 lesions identified for expert evaluation when capturing 80% and 90% of overall skin cancers, respectively. At these two thresholds, other ISIC’24 models were more precise despite a lower overall *AUC*. Put in terms of a patient-level perspective as opposed to the lesion-centric metrics, ISIC’24 models were observed to detect skin cancer in upwards of 79% of true-positive patients when triaging the most concerning 15 lesions per patient (highest overall *SE*_*top*-15_ = 0.7903) for clinical evaluation. These metrics are further detailed in the Methods section.

**Table 3:** Measures of diagnostic effectiveness across various automated approaches over the ISIC’24 private leaderboard evaluation dataset

Task	Model					Metric				
						*pAUC*_>80% *TPR*_	*AUC*	*NNT*_80% *SE*_	*NNT*_90% *SE*_	*SE*_*top*-15_
**Malignancy classification**	Best across all ISIC'24 submissions					0.173	0.968	42.26	88.60	0.790
	Marchetti et al.^a^					0.032	0.704	874.27	1013.24	0.360
	ISIC'24 winner - ablation variants	*Meta-basic*	*Meta-WB360*	*Tiles*	*Patient context*					
		x	x	x	x	0.173	0.967	50.57	98.20	0.729
		x	x	x		0.165	0.956	72.68	145.72	0.687
		x	x		x	0.164	0.957	63.62	167.16	0.695
		x	x			0.152	0.941	111.34	230.96	0.646
		x		x	x	0.161	0.948	97.79	165.34	0.597
		x		x		0.154	0.937	121.46	206.42	0.566
			x		x	0.157	0.949	85.36	193.69	0.644
			x			0.150	0.939	111.41	241.17	0.657
				x	x	0.142	0.923	160.88	306.20	0.544
				x		0.142	0.922	143.21	308.64	0.548
**Melanoma classification**	Best across all ISIC'24 submissions					0.176	0.970	126.31	261.92	0.791
	Marchetti et al.^a^					0.114	0.893	739.45	1610.18	0.541
	ISIC'24 winner - ablation variants	*Meta-basic*	*Meta-WB360*	*Tiles*	*Patient context*					
		x	x	x	x	0.169	0.962	212.36	412.60	0.689
		x	x	x		0.164	0.954	299.32	664.92	0.617
		x	x		x	0.168	0.961	156.65	505.10	0.708
		x	x			0.155	0.943	352.25	849.32	0.627
		x		x	x	0.163	0.949	338.96	487.13	0.605
		x		x		0.157	0.940	373.31	593.00	0.551
			x		x	0.159	0.950	344.88	595.41	0.614
			x			0.153	0.940	430.91	712.12	0.640
				x	x	0.152	0.934	435.42	804.10	0.541
				x		0.148	0.929	446.84	841.38	0.504

For each metric, the high score over all 4998 ISIC’24 submissions is reported. All model variants performed in the ablation study are summarized, including the winning model of ISIC’24, which utilized all four feature classes. The included feature classes are noted with an “x” for each model variant.

^a^Excluded 209 lesions from one patient who was included in both studies.

Model discrimination was similar whether classifying any form of skin cancer (including both melanocytic and nonmelanocytic types) or classifying melanoma specifically. In melanoma classification, ISIC’24 models scored as high as *pAUC*_>80% *TPR*_ = 0.1757, *AUC* = 0.9704, and *SE*_*top*-15_ = 0.7908. At the studied sensitivity thresholds and across multiple patients, ISIC’24 models would triage no fewer than 126 (*NNT*_80% *SE*_ = 126.31) and 262 (*NNT*_90% *SE*_ = 261.92) lesions for expert evaluation per detected melanoma. In comparison, the baseline melanoma prevalence in the evaluation set was 1 per 3744 lesions. These models outperformed the preliminary model for WB-based melanoma detection published by Marchetti et al. (*AUC* = 0.8927), where *NNT*_80% *SE*_ was 739.45.

**Fig. 2:**
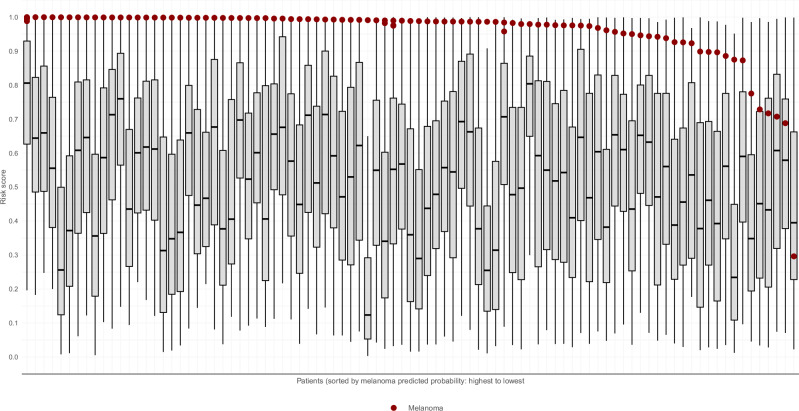
Lesion risk scores stratified by patient. Lesion risk score predictions by the winning model for each of the 91 patients whose tiles contained a melanoma. The risk score (y-axis) is displayed for melanomas (mean = 0.958, median = 0.988) as red dots, and all lesions (mean = 0.465, median = 0.437) as grey boxplots, stratified by each patient (x-axis). The patients are sorted by highest scoring melanoma. Lesion scores closer to 1 on the y-axis indicate higher model-estimated risk and lesions closer to 0 on the y-axis indicate lower model-estimated risk.

Distributions of the winning model predictions are presented in Fig. 2 for each of the 91 patients whose tiles contained a melanoma. 28% of melanomas were the highest-scoring lesion on the patient, 53% were in the top 99th percentile, and just 21% were below the 95^th^ percentile. The median (mean) number of lesions scoring higher than a melanoma was 5 (26). With the Marchetti et al. approach, 18% of melanomas scored the highest, 36% were in the top 99^th^ percentile, and 32% were below the 95th percentile of lesions on the patient.

### Relative importance of 3D TBP-based input feature classes on diagnostic performance

Extensive ablation studies were conducted to evaluate the relative importance of various input feature classes (i.e., tiles, WB360 “appearance” metadata, basic “demographics” metadata, and patient contextual information) on the diagnostic performance of the winning model. Notably, patient context (i.e., putting the lesion in the context of all other lesions from the given patient) had a substantial effect. When patient-contextual features were withheld from the full model, skin cancer discrimination was significantly lower (*AUC* = 0.956 vs. *AUC* = 0.967, *p* < 0.001) and, at the threshold of 80% sensitivity, triaged 22 additional non-malignant lesions (*NNT*_80% *SE*_ = 72.68 vs. *NNT*_80% *SE*_ = 50.57). Tiles were less informative than the pre-extracted WB360 measurements. The model variant restricted to WB360 “appearance” metadata significantly outperformed the variant that utilized only tiles (*AUC* = 0.939 vs. *AUC* = 0.922, *p* = 0.016), and the effect of excluding tiles from the full model was less detrimental than excluding the WB360 “appearance” metadata (*AUC* = 0.957 vs. *AUC* = 0.948, *p* = 0.068). Patient age, sex, lesion anatomical location, and hospital/institution label were also a critical factor. The inclusion of basic “demographics” metadata significantly improved the model variant that utilized only WB360 “appearance” metadata and patient context (*AUC* = 0.957 vs. *AUC* = 0.949, *p* = 0.003). All ablation study results are reported in Table 3.

### Association of lesion characteristics and ML-model perceived risk

Certain color measures were mildly-to-moderately correlated with mean ascending rank ordered risk score among the top-500 ISIC’24 models. Hue within the segmented lesion demonstrated the strongest association (*ρ* = −0.55) and was analogous to a spectrum of red versus brown; estimated risk tended to be higher in lesion-tiles with redder hue. Similarly, greater redness (lower hue) in the surrounding skin was also associated with a higher risk (*ρ* = −0.31). Lower average blue-yellow (b*) contrast between the lesion and outside skin (*ρ* = −0.47), higher color variance inside the lesion (*ρ* = 0.34), and higher color asymmetry (*ρ* = 0.34) inside the lesion also were among the color characteristics most strongly associated with risk estimates.

**Fig. 3:**
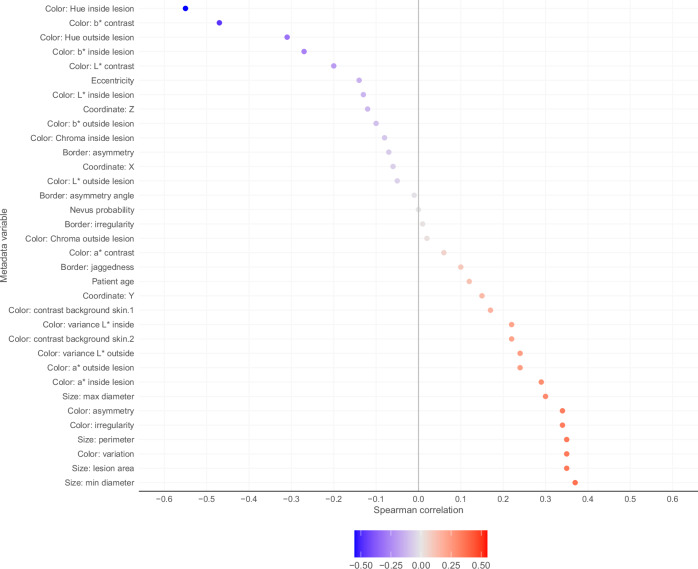
Association of lesion characteristics and ML-modelled risk. Waterfall graph presents correlations between each continuous metadata feature and the mean lesion risk score rank (ascending order) over the top 500 placing submissions of ISIC’24.

Measures of lesion size, including minor axis diameter (*ρ* = 0.37), lesion area (*ρ* = 0.35), and border perimeter (*ρ* = 0.35), showed a mild positive correlation with risk score. Conversely, border irregularity (*ρ* = 0.18) and asymmetry (*ρ* = 0.07) exhibited poor correlation with risk score. Figure 3 presents the degree of association between ML-modelled risk scores and each continuous-type metadata element.

## Discussion

ISIC’24 ranked among the most attended events on the competition platform, Kaggle. The competition was designed around the discrimination of skin cancer in patients who tended to have several hundred benign lesions. Systems that support cancer diagnostics are required to minimize false negatives, so participants were scored on a metric based on decision thresholds surpassing 80% sensitivity. In a dataset where just 1 in nearly 1100 lesions were malignant, the winning model could narrow that scope to 1 in 51 while triaging 80% of cancers, or 1 in 98 while triaging 90%. Dermatologists routinely examine hundreds of lesions per clinical patient, where prevalence is even lower still. These findings provide a proof of concept for new 3D TBP-based approaches to skin surveillance, which may help to streamline workflows in specialty clinics or improve referral of at-risk individuals.

Many participants submitted models achieving a similar performance to the winner, shown in the high distributional density towards the best scores on the leaderboard (Fig. 1). This, in part, explains the large jumps in rankings from the public to private leaderboards, a phenomenon typically referred to as “leaderboard shake-up” in ML competitions. However, there could be other factors that contributed to the shake-up. Some teams made hundreds of submissions to the public leaderboard for immediate feedback to iteratively optimize their algorithms. This method results in overfitting and often a gap between leaderboard scores. Another factor could be the limited number of diseased class examples, creating higher variability in submission scores. All three factors are likely to have contributed.

Leading ISIC’24 models utilized both images and metadata. Since the provided WB360 measurements were compiled from proprietary Vectra WB360 tooling, the leading models cannot be directly applied to external data without those measurements. Moreover, the winning model defined patient-contextual features, such as patient-wise normalization, to emphasize outlier lesions on a patient, mimicking clinical approaches like the Ugly Duckling sign^32–35^. Therefore, this model cannot be directly applied to analyze single lesions at a time.

In this respect, an ablation study was conducted to investigate versions of the winning model that depend on different combinations of input feature classes. The study defined four information classes—tiles, basic “demographic” metadata (i.e., patient age and sex, lesion anatomical location, and hospital/institution), WB360 “appearance” metadata (e.g., measures of lesion size, color, border irregularity, and contrast with background skin as well as lighting modality), and patient context—and measured their effects on diagnostic classification outcomes. The treatment of patient context and WB360 “appearance” metadata classes as experimental variables enabled the assessment of the feasibility of a single lesion analysis model, as well as applications that do not have access to WB360 measurements from the proprietary tooling.

**Fig. 4:**
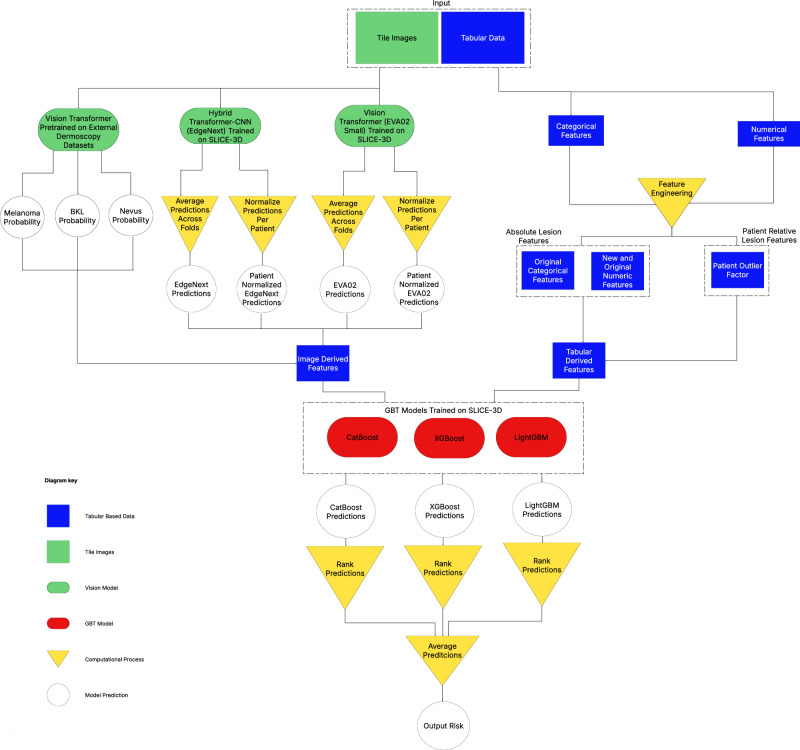
ML model diagram. Diagram of the winning model from the ISIC’24 competition, which was the subject to the ablation study.

The image-only models used by the winning algorithm were originally trained independently, without utilizing any metadata or patient-contextual information. These models were developed for different diagnostic classification tasks, as illustrated in Fig. 4. The winning model combined the probability estimates generated by these image-only models with metadata in the later stages to train boosting models that produce the final lesion risk scores. Given that the image models were trained independently of other features, it was deemed unnecessary to retrain them for each ablation variant. Conversely, the boosting models were retrained for each variant combination of image, metadata, and patient-contextual feature class, and the ablation study was carried out using these updated boosting models.

The ablation study underscores the limitations of the purely vision-based model. Image-only models should ideally be capable of extracting information related to lesion appearance from the standardized lesion tiles, eliminating the need for a separate model to extract the WB360-measurement features. However, the model variant trained only on WB360 “appearance” metadata outperformed the variant trained only on tiles. This indicates that these information classes complement each other, revealing that the black box feature extraction in vision models can lead to suboptimal solutions. This underscores the necessity for ongoing research aimed at enhancing the feature extraction process. In this context, emerging Vision Language Models will be essential in creating models designed to extract clinically relevant, explainable features from images. The basic “demographic” metadata feature class further improved diagnostic accuracy outcomes, which reinforces how clinically collected information can contribute meaningfully to ML-models. The value of multi-modal data is perhaps best demonstrated through the superiority of the model variant trained using all four input feature classes.

A novel result of this study is that it demonstrated the relevance of patient-context to an ML-model, underscoring an advantage of TBP imaging. Prior dermoscopic imaging datasets containing patient-clustered observations failed to contain rich phenotype information and skewed towards lesions of heightened clinical concern. Past efforts to develop models using those resources failed to effectively demonstrate the utility of patient-context^2,29^. One goal of the ablation study was to examine how patient context affects the diagnostic performance of the winning model. Ablation variants incorporating patient context features surpassed their independent-lesion equivalents at skin cancer discrimination (in terms of *AUC*), which reinforces the importance of considering patient norms. Still, single-lesion models have potential to be flexibly applied outside of TBP systems, and some model variants that did not utilize the patient context feature class still performed commendably well.

Although it did not perform as well as its counterparts that utilized metadata feature classes, the image-only model variant outperformed the pilot melanoma detection model by Marchetti et al. (*pAUC*_>80% *TPR*_ = 0.148 vs *pAUC*_>80% *TPR*_ = 0.114) and serves as a strong baseline in situations where collecting contextual or lesion appearance metadata is not feasible, such as when using a smartphone camera or conducting close-up clinical photography. In these cases, the images closely resemble the tiles utilized in this study. Moreover, the increased ability to discriminate skin cancer when adding readily available clinical data (i.e., basic “demographic” metadata) is encouraging (*pAUC*_>80% *TPR*_ = 0.154 vs *pAUC*_>80% *TPR*_ = 0.142).

The models generated from ISIC’24 demonstrated significant improvement over the pilot approach demonstrated by Marchetti et al.^27^ The winning model and each of its ablation variants performed better than the pilot model across all defined metrics. This likely reflects, in part, the impact of a larger training set, but also underscores the potential of higher-capacity ML-models over generalized linear models for triaging atypical skin lesions. Still, a promising aspect from the pilot model was that some of the pilot study results were replicated in this study. In terms of patient-specific percentile scores, 18% of melanomas in this study scored highest on the patient. Similarly, 14%^27^ scored highest in the pilot study.

Some extent of model interpretability is accentuated by analyzing associations between lesion appearance and mean (ascending rank-ordered) risk score (Fig. 3). Measures of lesion size and color variation were at least mildly associated with higher scores, which is in line with practical tools taught in clinical dermatology for identifying melanoma, such as the ABCD Checklist^36^ and the 7-Point Checklist^37,38^. However, border irregularity and asymmetrical shape are features typically linked with heightened clinical concern, but both exhibited poor correlation with risk scores (*ρ* = 0.01 for border irregularity, *ρ* = -0.07 for border asymmetry). The dataset also included non-melanocytic lesions that lack pigment, and red lesions (lower hue) tended to be ascribed to a higher risk than brown lesions (higher hue). Actinic keratoses appear scaly and red, occur commonly in individuals after chronic sun exposure, and are generally viewed as potential precursors to SCC. However, risk score distributions were not statistically different between the biopsy-proven actinic keratoses and SCCs (*p* = 0.906, Kolmogorov–Smirov test). Future efforts to improve diagnostic performance in non-melanocytic lesions may provide a significant impact on automated and semi-automated TBP-based skin cancer detection.

The datasets in this study were sourced from multiple centers, each with distinct patient phenotypes and varying applications of 3D TBP imaging. Notably, the propensity for cross-polarized light versus white light was not consistent across centers^23^. Lighting impacts the visibility of lesions on 3D TBP photography, and pigmented lesions are more readily detected under cross-polarized lighting. Therefore, variation in patient lesion counts between hospitals can be attributed to technical settings as well as patient phenotypes, which complicates comparisons of model discrimination from one center to another. It is imperative that future research evaluates the generalizability of 3D TBP-based ML-models to uphold fairness and reliability across diverse patient populations. Furthermore, ISIC’24 models used hospital labels to inform predictions in the evaluation set that do not describe all potential use settings. Prior to implementing these models in new contexts, it is crucial to reassess their performance with local patient samples and consider necessary recalibrations.

There are several barriers to applying ISIC’24 models for 3D TBP-based atypical lesion triage. First, the technology relies on 3D TBP imaging, which remains less accessible and more expensive than standard clinical and dermoscopic imaging methods. Additionally, the model’s performance relies on specific lesion appearance features derived from Vectra WB360’s proprietary algorithm, complicating its applicability across different imaging systems. Furthermore, model efficiency is another important factor. For instance, a preliminary trial of the winning model demonstrated processing times of 70 s on a GPU and 390 s on a CPU per 3D TBP capture. Although time constraints were established for submissions to ISIC’24, it remains essential to evaluate what processing times are deemed acceptable by clinicians and the implications for real-world application.

ISIC’24 improved on the framework laid by Marchetti et al.^27^ by delivering models that improved precision using multi-modal data. In a dataset with a prevalence of 0.09% (342 skin cancers in 370,704 lesions), the winning algorithm could lower the number of lesions needing expert assessment by 95% or 91% while identifying 80% or 90% of true positives, respectively. These results provide evidence that 3D TBP-based applications may be effective in performing atypical lesion triage. Further clinical studies are essential to evaluate the reliability of these models as well as to determine appropriate thresholds, which may need to be tailored to unique individuals. Aside from model accuracy, costs^39^ should also be considered. This includes economic factors as well as overtreatment, as introducing a new technology for clinical triage or screening has the potential to contribute to overdiagnosis^40,41^. Therefore, the overall clinical utility of 3D TBP-based triaging applications should undergo rigorous testing.

## Methods

### ISIC’24 competition and dataset

An online competition called “ISIC 2024 – Skin Cancer Detection with 3D-TBP” (ISIC’24) was held on Kaggle, a data science platform, from June 27th through September 6th, 2024. Participants submitted ML-based risk-prediction models that distinguish pathology-confirmed skin cancer among candidate lesions identified on Vectra WB360 images. The official training dataset was the previously described and publicly available SLICE-3D dataset^23^, compiled from seven medical centers across North America, Europe, and Australia. In summary, the training dataset consisted of about 400,000 lesion tiles that were cropped from 3D TBP images of about 1000 patients and extracted in JPEG format. Each tile encompassed 15 mm-by-15 mm areas of skin centered on distinct lesions identified by Vectra WB360 lesion-detection tooling. Metadata was provided, including “basic metadata,” “WB360-measurements,” and diagnostic labels, as described by Kurtansky et al.^23^. Basic metadata are obtained from patient profiles, such as age, sex, lesion location, and hospital, which and are not directly attained from images. WB360-measurements characterize lesion appearance, like size, color, and border irregularity, and are estimated solely from 3D TBP images by proprietary Vectra WB360 tooling. Although the competition was designed as a binary classification challenge, the training metadata also contained a granular diagnosis. Disease-positive examples included invasive or in situ melanoma, basal cell carcinoma, and squamous cell carcinoma. Disease negative examples included lesions that were not biopsied, many of which appear clinically as nevi and lentigines, and other pathology-confirmed cases such as benign or indeterminate melanocytic proliferations, vascular lesions, fibromas, benign keratoses, and actinic keratoses. The decision to include actinic keratoses and indeterminate melanocytic lesions in the disease-negative class was based on consensus among the competition organizers who practice dermatology, despite a heightened risk of developing subsequent skin cancers^42–44^. There were no known duplicated lesions in the dataset; all qualified^23^ lesions larger than 2.5 mm, and any that were later biopsied, were included from each patient. The participants were also allowed to use publicly available external data. Competing submissions were judged on test data compiled from the same medical centers (albeit different patients than the training dataset), plus from two additional sources. Methods of patient, lesion, and image selection followed the previously described approach^23^. Test data comprised about 500,000 lesion tiles from more than 1200 patients. The same metadata elements were provided except that diagnosis characteristics were excluded, including the ground truth label (disease positive vs disease negative).

Participants contended for $80,000 in prizes. The primary scoring metric was the partial area under the receiver operating characteristic (ROC) curve above an 80% true positive rate (*pAUC*_>80% *TPR*_), with possible values ranging from 0.0 to 0.2. To mitigate the possibility of human labeling (“hand labeling”) to achieve higher scores, participants were prohibited from accessing the test set directly. Instead, participants submitted code solutions in the format of Kaggle notebooks to be executed on the Kaggle platform, where they accessed the hidden test set and yielded prediction outputs that were saved internally and used for scoring. Notebooks were required to finish executing within 12 h. The test dataset was further partitioned into public and private leaderboard subsets to mitigate result probing while offering an interim benchmark. There was no patient overlap across subsets, with 70% of patients allocated to the private leaderboard subset. Participants could submit up to five entries per day for immediate scoring on the public leaderboard. Participants were allowed to pick two of their submissions for the official scoring on the private leaderboard, which was disclosed after the final submission deadline. Prize-winners were required to submit their code and documentation for both model training and prediction.

Participants agreed to the Kaggle competition rules permitting the use of their submissions for research and publication. Access and use of image datasets adhered to the Terms and Conditions of the ISIC Archive. The respective medical institutions submitted data to the ISIC Archive that was duly authorized to be transmit and licensed. The Alfred Hospital Ethics Committee granted approval (746/23) for contribution of de-identified ACEMID^16^ data, which was acquired with informed consent. Written informed consent was obtained at FNQH Cairns. Consent was waived for use of anonymized images at Memorial Sloan Kettering Cancer Center (Institutional Review Board Protocol 16-974). The research ethics committee of the Hospital Clinic de Barcelona approved (HCB/2023/0213) the licensing of the data, which was collected with written consent for use of anonymized images for scientific purposes. Data from the University of Queensland Dermatology Research Centre were collected in various studies with written consent and approved for contribution by Metro South Human Research Ethics Committee (HREC) (HREC/16/QPAH/125 and HREC/17/QPAH/816), University of Queensland HREC (2016000554 and 2018000074), the Queensland University of Technology (1600000515), and QIMR Berghofer Medical Research Institute (P2271). The ethics committee of the Medical University of Vienna approved (1996/2023) the use of pseudonymized images for scientific purposes, which were collected with written consent. The research ethics committee of Andreas Sygros Hospital approved (MSK.DTA.0000.0582) the licensing of anonymized images that had been collected with written consent for scientific purposes. Melanoma Institute Australia received approval from the Sydney Local Health District HREC (X20-0241 and 2020/ETH01411: Melanoma Image Annotation and Analysis Collaboration) to share ACEMID^16^ data, which was acquired with informed consent. Data from the University Hospital Basel were acquired with written consent for publication and transferring of data as part of a trial approved by the local ethics committee in Switzerland (2020-02482) and registered with ClinicalTrials.gov (NCT04605822).

### Evaluation of ML-models

Officially scored ISIC’24 submission outputs were acquired to further study the diagnostic performance of TBP-based ML-models. Measures of diagnostic discrimination included *pAUC*_>80% *TPR*_ and area under the full ROC curve (*AUC*). To gauge performance in theoretical triaging scenarios, two additional metrics were defined: *SE*_*top*-15_ and *NNT*_*x*% *SE*_. *SE*_*top*-15_ measured sensitivity under the hypothetical task of identifying 15 lesions with the highest risk scores on each patient. This patient-centric metric was used as a secondary prize metric in ISIC’24 to conceptualize how full-body skin examinations may be supported by automated lesion selection. The computation of *SE*_*top*-15_ weighed each diseased patient equally to avoid being more strongly influenced by patients who had multiple malignancies. *NNT*_*x*% *SE*_ defined the average number of lesions needed to triage to undergo expert evaluation to detect a single malignancy, using a threshold corresponding to a given sensitivity based on the private leaderboard set. This precision-based metric is similar to the number needed to biopsy to detect melanoma (NNB)^45^, which is used in dermatology to measure the trade-off between skin cancer detection and avoidable interventions. Two test decision thresholds are considered by reporting both *NNT*_80% *SE*_ and *NNT*_90% *SE*_. Reports of diagnostic performance were computed using model prediction outputs restricted to the private leaderboard set.

Associations between lesion characteristics and estimated risk across top-performing models were measured to study qualities of perceived risk. First, the 500 highest-placing submissions were filtered to the private leaderboard set. Due to variation in output distributions, each submission was normalized through rank (ascending order) transform. The mean rank for each tile was computed, yielding a summary score for model-estimated lesion risk. Associations with continuous features were measured with Spearman’s correlation coefficient.

### Ablation study

The solution that placed 1st in ISIC’24 was selected for a post-hoc ablation study to evaluate the influence of key features on diagnostic performance. The experiment incorporated both image and metadata features (Table 1), and the model architecture is depicted in Fig. 4. Its image processing branch comprises an ensemble of three neural network classification models: Two EVA models^46^ and one EdgeNext^47^. The first EVA model is trained on external dermoscopy data, while the EdgeNext and second EVA model are trained independently on tiles exclusively. Five versions of each model were trained with cross-validation, where 80% of the training data was used for training and the remaining 20% for validation. For each fold, final parameters were determined by highest performance on the set-aside validation set. Aggregation occurs over the five folds of each respective model. The metadata processing branch utilizes all available metadata, including basic “demographics” and WB360 “appearance” metadata. The pipeline also defines interaction terms and “patient-contextual” terms, which are those derived from other examples involving the same patient. Patient-contextual features include normalized terms (for instance, lesion diameter adjusted against the mean of the patient) and patient phenotype summaries (for instance, total lesion count). Some patient-contextual features mimic approaches to clinical outlier lesion detection, like the ugly duckling sign^32–35^. The neural network outputs and metadata features are fed into three gradient boosting tree (GBT) models whose outputs are aggregated to generate a risk estimate for each lesion.

The ISIC’24 winning model was reproduced in a local environment, confirming the validity of the original solution and feasibility of an ablation study. Input feature elements were categorized into three feature classes: (1) tiles, (2) basic “demographics” metadata (i.e., patient age and sex, lesion anatomical site, and hospital), (3) WB360 “appearance” metadata. These classes were used to create ten variants of the model: (1) all feature classes, (2) tile images only, (3) tile images with basic metadata, (4) WB360 metadata only, and (5) WB360 metadata with basic metadata. Additionally, a fourth feature class represented (4) patient-contextual features derived from having multiple observations from the same patient. Each experiment was conducted with and without the component of patient-contextual feature class. Table 3 displays the ten model variants of the ablation study.

The statistical model by Marchetti et al.^27^ was used as a benchmark of diagnostic performance. Their multivariate model used 11 morphological WB360 measurements (i.e., lesion area, border jaggedness, lightness contrast between lesion and background skin, red-green contrast between lesion and background skin, blue-yellow contrast between lesion and background skin, WB360-defined color contrast, minimum lesion diameter, color variation, color asymmetry, variance of lightness in background skin, and asymmetry angle) and five categories of anatomic site (i.e., head or neck, anterior torso, posterior torso, upper extremity, and lower extremity) as independent predictive factors. Model coefficients were acquired from the authors. The model was evaluated over the ISIC’24 private leaderboard set, minus one patient who was also included in the study by Marchetti et al.^27^.

Two-sided DeLong’s test was used to statistically compare model variants in discriminating skin cancer. The chosen level of significance was 0.05. The ablation study experiments were performed using Python (version 3.8.20). Statistical analyses were performed using R Statistical Software (version 4.0.3).

## Data Availability

The training dataset^23^ used in ISIC’24 is available on the ISIC Archive and at [10.34970/2024-slice-3d](10.34970/2024-slice-3d). The ISIC’24 evaluation dataset files will remain private indefinitely to support future ISIC competitions. However, individuals may use the late submission system^31^ on Kaggle to submit their models and benchmark scores against the ISIC’24 competition leaderboard.

## Code availability

The description of the winning model is publicly available at the competition page on Kaggle^31^ at https://www.kaggle.com/competitions/isic-2024-challenge/discussion/533196 and the source code is available on GitHub at https://github.com/ilyanovo/isic-2024/tree/main. The model variants of the ablation study are available at https://github.com/mauragillis/ablation-study.
